# A longitudinal inter-vehicle distance controller application for autonomous vehicle platoons

**DOI:** 10.7717/peerj-cs.990

**Published:** 2022-05-23

**Authors:** Alex Gunagwera, Aydin Tarik Zengin

**Affiliations:** Department of Computer Engineering, Istanbul Sabahattin Zaim University, Kucukcekmece, Istanbul, Turkey

**Keywords:** Autonomous vehicles, Autonomous vehicle platoons, Intelligent transport systems, Longitudinal platoon control, Automated highway systems, PID, Communication

## Abstract

Autonomous vehicle platoons are a promising solution to road safety, efficient road utilization, emission reduction, among other problems facing today’s transportation industry. However, consistently maintaining the desired inter-vehicle distance is one of the major problems facing autonomous vehicle platoons. In this study, we propose a proportional–integral–derivative (PID)-based cost-efficient algorithm to control the longitudinal inter-vehicle distance between successive members of an autonomous vehicle platoon. In our approach, calculations of the control algorithm are decentralized, and the data used in the control algorithm is obtained using one sensor per platoon member making the algorithm cost-efficient both computationally and financially. The proposed algorithm was implemented using the Robot Operating System (ROS) and applied to 3D vehicle models in simulations designed to mimic the natural environment in order to demonstrate and evaluate the suitability of the proposed algorithm for demanding and applicable scenarios. We performed meticulous simulations using the ROS framework in conjunction with the gazebo platform. In the proposed approach, the desired inter-vehicle distance between platoon members was successfully kept with a maximum absolute error of 5 m under any given scenario at any given time while maintaining platoon formation and ensuring that no collisions occur among platoon members.

## Introduction

Autonomous vehicles (AVs) have been a dream for over 80 years, but it was not until the 1990s that significant progress in the field emerged. The most prominent being General Motors’ working prototype presented in the 1960s (Rannenberg, 2016; Herman, 2012). Consequently, Autonomous Vehicle Platoons (AVPs) became a trending topic. Platoon driving refers to having more than one vehicles linked and traveling together as a single unit with the same lateral and, or longitudinal motion control.

Autonomous Vehicle Platooning yields various benefits ranging from efficient road capacity utilization (Janssen et al., 2015), reducing the workload of employees (Kimura et al., 2017), minimizing traffic accidents resulting from human error (Belcher et al., 2018), to saving the time spent on driving.

A plethora of studies on Vehicle Platoons, Autonomous Vehicle Platoons, and their control have been carried out. For instance, studies on Adaptive Cruise Control (ACC) by Sivaji & Sailaja (2013), Cooperative Adaptive Cruise Control (CACC) (Shladover et al., 2015; Naus et al., 2010), String Stability (Swaroop, 1996; Cremer, 1992; Seiler, Pant & Hedrick, 2004; Swaroop et al., 1994). The cruise control model proposed by Sivaji & Sailaja (2013) was implemented in MATLAB SIMULINK. They used the velocity and inter-vehicle distance deviation as inputs to their controller.

The quality, ease of communication and type of information shared significantly affects the performance of an autonomous vehicle platoon. Furthermore, the efficiency of the communication methodology employed and the amount of information communicated in the platoon also enhance the performance and success of the entire platoon. Shladover et al. (2015) provided the essential definitions and distinctions among the different types of CACC and the various communication types employed by platoons. Robust Vehicle to Vehicle (V2V) communication, such as VANET, DSRC presented in Willke, Tientrakool & Maxemchuk (2009), Jain & Saxena (2017), and Vehicle to other Infrastructure (V2X) communication facilitates the functioning of AV platooning. Naus et al. (2010) presented a CACC technique whose string stability behavior was evaluated using a frequency-domain-based approach. They further verified the obtained theoretical results using practical experiments and analyzed the string stable and unstable behavior. Mei et al. (2018) investigated the optimization of joint radio resource allocation in LTE-V2V networks. Shen et al. (2018) discussed the effects of communication reliability and latency on the performance of vehicle systems using 5G V2X hardware prototypes and the 802.11 communication protocol. In our study, we utilize the Robot Operating System (ROS) (Quigley, 2009), for communication within the platoon. Inter-vehicle communication is wireless and is based on Wi-Fi (IEEE 802.11) network.

String stability is one of the most critical aspects of an autonomous vehicle platoon. Swaroop (1996) introduced and formally defined the notion of string stability, in general, pertaining to a system of countably infinite non-linear systems. He further analyzed the conditions under which string stability of such systems could be achieved. Cremer (1992) provided a string stability criteria depending on the error in velocity of each vehicle in comparison to the velocity of the Leading Vehicle (*LV*). His standards did not rely on the inter-vehicle distance. As far as autonomous vehicle platoons are concerned, string stability ensures that the inter-vehicle distance and velocity errors of platoon members do not grow as one moves up the platoon (Ghasemi, Kazemi & Azadi, 2013). Additionally, Xiao & Gao (2011) investigated the string stability of homogeneous and heterogeneous vehicle platoons with constant time headway spacing while considering the time delays in sensors and actuators. Seiler, Pant & Hedrick (2004) defined platoon stability as the error between the desired and the actual inter-vehicle spacing. Swaroop et al. (1994) presented string stability requirements that depend on the inter-vehicle distance in two categories-the strong sense and the weak sense. In the strong sense, the given string stability conditions require that the maximum inter-vehicle distance error of the *i*^*th*^ vehicle should either be equal or less to that of the *i* − 1^*th*^ vehicle. String stability in a weak sense has a requirement that just the maximum inter-vehicle distance errors should be less than or equal to those of the first follower (*F*) vehicle.

The Global Positioning System (GPS) (Edelkamp, Jabbar & Willhalm, 2003) is one of the most valuable sensors in look-ahead systems. For example, Sukkarieh, Nebot & Durrant-Whyte (1999) illustrated a high integrity navigation system’s development and implementation for usage in autonomous land vehicle applications. They mainly used GPS and the Inertial Measurement Unit (IMU) in their work. GPS is the backbone of the approach we present in this study.

Owing to the fact that AVPs have got to perform and work in mixed traffic currently, and the transition to fully autonomous vehicle platoons on highways in production is a gradual process, numerous studies tackled the issue of Human-Vehicle interaction and cooperative platoon driving. For instance, Kimura et al. (2017) discussed a partially autonomous platoon system that operates indoors and in relatively narrower environments. In their system, the platoon comprises a virtual autonomous leading vehicle projected on a head-mounted display and a real follower vehicle monitored by an assisted human driver. We believe that with modifications such as the installation of position beacons (Siegwart & Nourbakhsh, 2004), in the indoor or narrower target environments, the proposed control approach in this literature is a promising alternative in similar scenarios and settings that could contribute towards the achievement of complete autonomy of a vehicle platoon system.

Ali, Popov & Charles (2010) explored platoon performance when significantly large decelerations are applied. Swaroop & Hedrick (1999) discussed and analyzed various approaches to constant spacing control (constant headway) strategies. Rajamani (2011) and Girard, Spry & Hedrick (2005) investigated variable spacing control techniques. Ferrara & Vecchio (2007) studied the effect of local communication among vehicles and further proposed various collision avoidance methods between controlled vehicles and vulnerable road users.

Inter-vehicle distance, inter-changeably referred to as the inter-vehicle gap in this study, is an essential metric in autonomous vehicle platoons. How precisely inter-vehicle distance is kept and maintained ensures safe, comfortable, and more efficient road usage, among others. It is with this background that we propose this study.

In this paper, we propose a cost-efficient, proportional-integral-derivative (PID)-based algorithm for controlling the inter-vehicle distance between successive members of an autonomous vehicle platoon. Our approach differs from other numerous studies mainly by using only the onboard GPS sensors of the vehicles, gazebo robot simulator, and the robot operating system (ROS). Furthermore, our PID controller only requires the updated longitudinal inter-vehicle distance to the preceding vehicle and the platoon Leading Vehicle (*LV*), unlike most PID approaches that require the velocity and, or acceleration information as well. The algorithm observes maintenance of platoon formation and makes sure no collisions occur amongst platoon members. Obtained results are presented, and simulations of the system using 3D vehicles are further carried out using ROS and Gazebo platforms to verify the performance of the proposed approach.

The rest of this paper is organized as follows: The “experimental and environmental setup” section presents the setup of the experiment and the environment in which we carry out the experiments and simulations. The “Platoon longitudinal inter-vehicle distance control and problem statement” section explains the main problem tackled in this work and provides the main problem formulation. The “Results” section presents the results obtained from the simulations, whereas in the “Discussion” section, we discuss the obtained results, the limitations of the proposed approach, the applicability of the proposed algorithm. Furthermore, how the proposed algorithm differs from current related works. Finally, in the “Conclusion” section we summarize this work and present directions for future work.

## Experimental and Environmental Setup

3D vehicles were designed and modeled using gazebo and ROS environments. Visuals of the 3D vehicle objects were created using the gazebo platform, whereas vehicle motion was handled and controlled by the nodes implemented using the ROS framework (Quigley, 2009).

**Figure 1:**
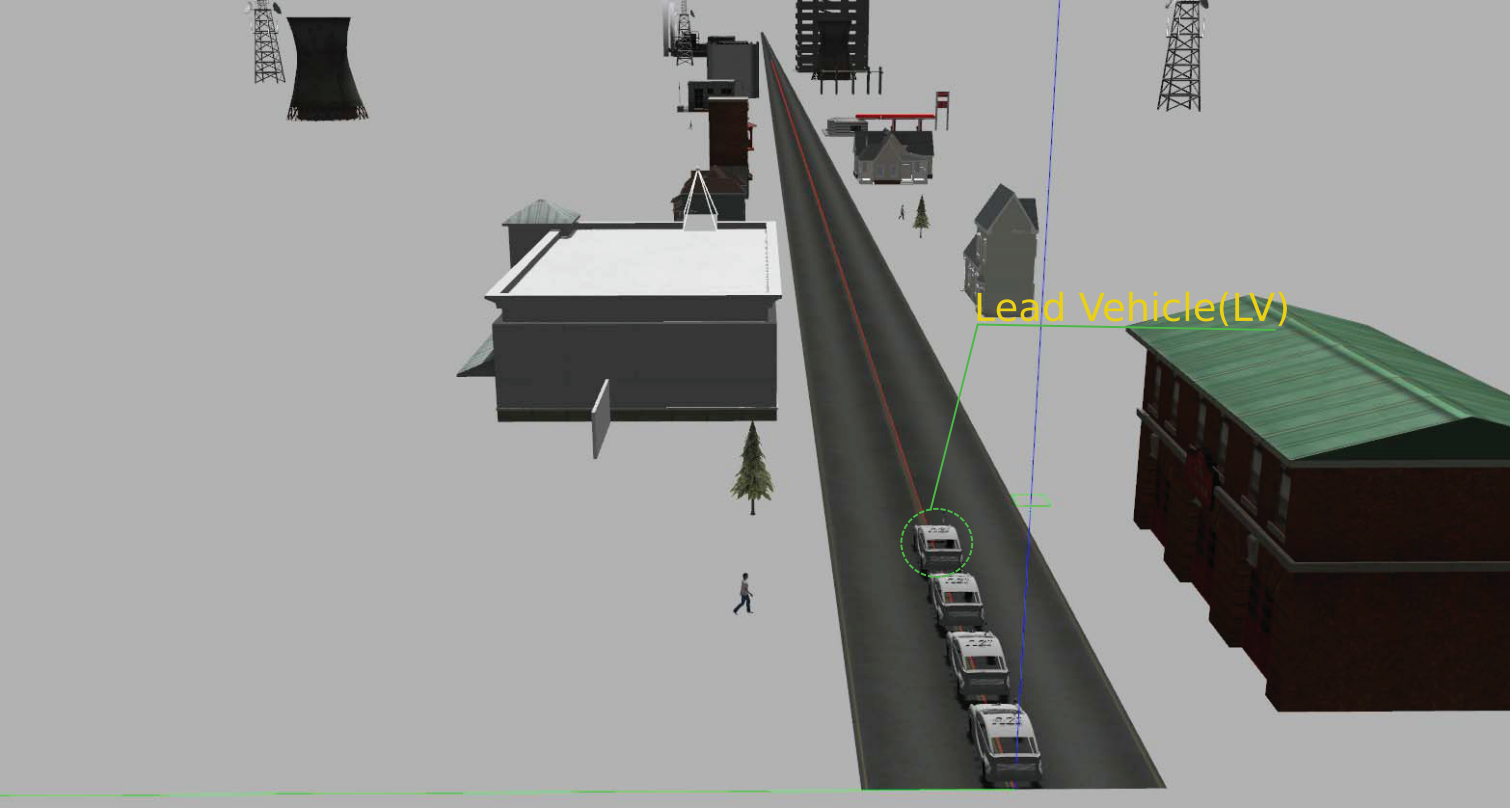
Simulation environment.

Figure 1 shows the environment in which the simulations were performed and monitored. The vehicles move forward along the road during simulation to preserve the presented platoon formation and the desired inter-vehicle distance.

We performed simulations under the assumptions and constraints that:

- the roads are straight and have got no slope so that longitudinal control of the platoon remains the focus of the platoon.
- communication is wireless, that is, over Wi-Fi (IEEE 802.11), and each vehicle is only allowed to communicate to the preceding vehicle.
- overtake and reversing maneuvers are not allowed in the platoon.

The platoon comprises four vehicles with-not obligatorily-homogeneous attributes. The vehicle attributes are presented in Table 1. We approximated these attributes in reference to a small vehicle such as the Hyundai Genesis-2014 presented by Edmunds (2014).

**Table 1:** Vehicle information.

Vehicle attribute	Value
Vehicle mass	1,823.0 kg
Vehicle length	5 m
Vehicle width	1.89 m
Vehicle height	1.480 m
Wheel radius	0.34 m
Wheel base	2.95 m
Wheel width	0.225 m
Drag coefficient	0.27 cd

The vehicle attributes are used in the description of the dynamics and kinematics of the joints that ultimately make up the vehicle model. ROS obtains dynamic and kinematic information of the robot by parsing Universal Robot Description Files/Formats (URDF) (Newman, 2017), which are based on the Extensible Markup Language (XML). From this information, ROS conveniently calculates and generates a robot description and stores it in the ROS parameter server. From this server, all information concerning the robot is then available for processing and manipulation. A robot is defined as a set of rigid kinematic links connected by joints in a 3 dimensional (3D) world within the URDF files. The joints connecting the robot links can be one of the following six types (Angeli, 2018):

- planar - allows motion in a plane perpendicular to the axis.
- floating - allows motion for all six degrees of freedom.
- continuous - a hinge joint; rotates around the axis with no upper and lower limits.
- fixed - not an actual joint since it cannot move. All degrees of freedom are locked. Its configuration requires not the axis, calibration, dynamics, safety control, or limits.
- prismatic - a joint that slides along the axis and has a limited range specified by upper and lower limits.

Alternatively, ROS provides the XML Macro (XACRO) files which enable the construction of shorter, more readable, and easier to manage XML files. XACROs can perform essential arithmetic evaluations. They provide the feature of code reuse since they can access macros from external files-so xacro files have the ability to extend other xacro files. This feature simplifies the modeling of more complex robots *e.g.*, vehicle parts; wheel, body, steering, etc., can be described in individual xacro files, and then combined in a single xacro file, say *vehicle.xacro*. The *vehicle.xacro* file is then expanded to generate the final URDF file used by ROS. ROS stores the robot information as a robot description in the ROS parameter server from which one can access this information using any higher-level programming language supported by ROS. Such languages include; Python, C++ to mention but a few. Furthermore, the xacro files are also capable of parsing arguments defined in YAML configuration files (Conley, 2009). The ability to parse arguments enables the use of variables in the evaluation of arithmetic operations. We pass the vehicle attributes presented in Table 1 as arguments to the xacro files since this format allows freedom of modification for the various vehicles, *i.e*., we can spawn simultaneously spawn multiple robots with similar or different attributes using the same robot template with varying values.

**Figure 2:**
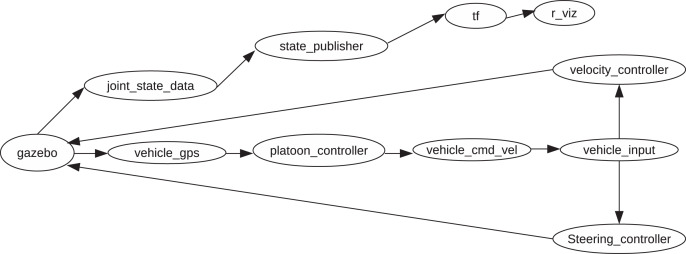
Internode relationships.

Gazebo, (Koenig & Howard, 2004), is an open-source robotics simulator developed by the University of California and Willow Garage. ROS and gazebo interact intimately to facilitate robot control and simulation. Gazebo offers an option to launch the simulator as a ROS node. Thus, information published *via* ROS messages and topics becomes available for utilization to the gazebo simulator. Figure 2 shows the generalized structure of the nodes and their connections. The *platoon*_*controller* node is the center of our inter-vehicle distance control algorithm. Within this node is the PID control algorithm which takes as input the GPS data containing coordinate measurements of the preceding vehicle and the *LV*. The *F* vehicle running the algorithm then calculates its distance to the preceding vehicle and the *LV* and passes the result to the PID control algorithm as the control signals. The joint output of the PID controller(s) becomes the velocity reference of the *F* vehicle in question. GPS data is generated by a GPS sensor attached to each vehicle, and the data is published by every vehicle’s corresponding *vehicle*_*gps* node. The *F* vehicle only needs to subscribe to the topic to which the preceding vehicle publishes its data. Each vehicle knows its node name, and all data is *namespaced* with the vehicle node name followed by the topic name to which the data is published. *E.g*., the *LV* (first vehicle of the platoon) is named *vehicle*1. Its GPS data is published to the topic named *vehicle*1/*gps*. The vehicle immediately behind the *LV* is named *vehicle*2 and its corresponding GPS data is published to *vehicle*2/*gps* and so forth. Assigning names to vehicle node names occurs during the platoon formation process; during the generation of the vehicle description information for ROS, and right before the generated robot models spawn in the gazebo simulator.

The *platoon*_*controller* publishes PID output velocity references to the respective *vehicle*_*cmd*_*vel* nodes of the corresponding vehicles *via* separate *vehicle*_*cmd*_*vel* topics. The *vehicle*_*input* node consumes this velocity reference data and prepares the different velocity components from this data, *i.e*., either angular or linear velocity. The *steering*_*controller* node handles the angular velocity component, whereas the *velocity*_*controller* node handles the linear velocity component. Equations (4) and (5) provide the main formulae which we employ to calculate the velocity of the vehicles.

*ROS control* is a rather helpful ROS package utility for robot control in ROS. It provides ready implemented joint control loops such as PID loop, Sinesweep to ensure joint frequency control, to mention but a few. The utility further aids in the abstraction of the robot hardware through hardware interfaces for all the uniquely defined robot joints. The *velocity*_*controller* and *steering*_*controller* nodes implement the interfaces and provide the inputs to the ROS control utility. This utility abstracts the direct interaction of our application with the vehicle resources such as actuators/motors, sensors, etc. Chitta et al. (2017) provide more information about the details of the utility’s method of operation, configuration, and the like. The *r*_*viz*, *state*_*publisher*, *joint*_*state*_*data*, and the *tf* nodes are important utility nodes. The *r*_*viz* node is from the *r*_*viz* ROS package, which enables robot visualization in real-time. Using this node, we can check the status of each vehicle. The *joint*_*state*_*data* node holds data of the joints of the robot. We can monitor joint states at any given time. This data is made available by the ROS control package. The *state*_*publisher* node enables us to publish this data as we wish. The *tf* node provides access to functionalities offered by the Transform library *TF* (Foote, 2013). This library provides a standard way to track data in different 3D coordinate frames in real-time since robots generally have multiple constantly changing coordinate frames over time. These frames include the world frame, base frame, etc. This node makes it possible for us to track any robot frame we are interested in at any moment in time. The *gazebo* node facilitates connection to the gazebo simulator, which we use to visualize the entire platoon during simulation.

Equations (1)–(3) represent the inertia matrices of the vehicle main body, vehicle wheel and the vehicle steering as defined in the xacro files which are ultimately evaluated to derive the underlying lower-level vehicle dynamics:

(1) $$VehicleBodyInertia = \left[ {\matrix{ {\displaystyle{{({W^2} + {M_h}^2) \cdot M} \over {12}}} & 0 & 0 \cr 0 & {\displaystyle{{({L^2} + {M_l}^2) \cdot M} \over {12}}} & 0 \cr 0 & 0 & {\displaystyle{{({W^2} + {L^2}) \cdot M} \over {12}}} \cr } } \right]$$

(2) $$WheelInertia = \left[ {\matrix{ {\displaystyle{{M \cdot {R^2}} \over 2}} & 0 & 0 \cr 0 & {\displaystyle{{{R^2} \cdot M} \over 4} + \displaystyle{{{W^2} \cdot M} \over {12}}} & 0 \cr 0 & 0 & {\displaystyle{{{R^2} \cdot M} \over 4} + \displaystyle{{{W^2} \cdot M} \over {12}}} \cr } } \right]$$

(3) $$SteeringInertia = \left[ {\matrix{ {\displaystyle{{{R^2} \cdot M} \over 4} + \displaystyle{{{L^2} \cdot M} \over {12}}} & 0 & 0 \cr 0 & {\displaystyle{{M \cdot {R^2}} \over 2}} & 0 \cr 0 & 0 & {\displaystyle{{{R^2} \cdot M} \over 4} + \displaystyle{{{L^2} \cdot M} \over {12}}} \cr } } \right]$$

where: *W* is the vehicle width, *M*_*h*_ is vehicle height, *L* is the vehicle length, *M* is the vehicle mass and *R* is the wheel radius. We use such matrices to also define the limits and values of the vehicle joints in to have the 3D vehicle models spawned in the Gazebo platform move realistically in our 3D world. Further details of low-level designs and control are left out for brevity since they are not the main focus of this study.

We calculate the velocity to the rear left and right wheel actuators as follows:

(4) $${V_{rb}},{V_{lb}} = f({v_x},r) = {v_x}/r$$

Equation (4) yields the velocity components for the left (*V*_*lb*_) and right (*V*_*rb*_) rear wheels of the vehicle. Where *v*_*x*_ is the *x* component of the linear input velocity, and *r* is the radius of the vehicle’s wheel.

Finally:

(5) $$V = \displaystyle{{{V_{rb}} + {V_{lb}}} \over 2} \cdot r$$

*V* is published to a PID controller whose result is then directly issued to the joint-links controlling the vehicle wheels. Control of steering is beyond the scope of this study and thus left out for brevity.

Initially, all vehicles are at rest (0 m/s) and are at an inter-vehicle distance of 1 m apart. The inter-vehicle distance is measured from the head of the following vehicle to the rear of the preceding vehicle. The GPS sensors are, however, placed at the corresponding vehicle’s center of mass as (Redondo et al., 2018) recommend. The *LV* starts accelerating uniformly at 0.1 m/s^2^ at a time, *t* > 0 s with a time-step of 0.1 s until it finally reaches a pre-defined velocity reference, *V*_1_ = 22 *±* 0.001 m/s. It then moves with constant velocity, *V*_1_ m/s for a pre-defined duration, *T*_1_ ≈ 55 s, after which period the *LV* starts to gradually decelerate at a rate of 0.1 m/s^2^ at time intervals of 0.1 s until it reaches velocity *V*_2_ = 10 *±* 0.001 m/s. The *LV* moves with constant velocity, *V*_2_ m/s for a duration of *T*_2_ ≈ 74 s, after which it linearly decelerates to velocity *V*_3_ = 5 *±* 0.001 m/s and maintains *V*_3_ for *T*_3_ = 74 s. Ultimately, the *LV* decelerates to rest. We terminate the simulation after the entire platoon reaches 0 m/s, *i.e*., at rest. The follower (*F*) vehicles accelerate and decelerate accordingly, with their reference velocities being the joint outputs returned by their respective PID controllers. Each *F* vehicle simultaneously controls its distance to both the preceding vehicle and the *LV*-except the second *F* vehicle whose preceding vehicle happens to be the *LV*. We, additionally, include a different scenario during which the *LV’s* velocity is uncertain and changes continuously. That is, the velocity of the *LV* is never constant. Given that acceleration and deceleration of the *LV* can be viewed as disturbances in a platoon according to (Zheng et al., 2014) the LV occassionally exhibits random, abrupt and sharp accelerations and/or decelerations during this scenario. This behavior is intended to simulate miscellaneous real-life occassions where the *LV* maybe required to make an abrupt deceleration or acceleration For instance, to either dodge an obstacle or prevent collision. At the end of this scenario, the LV decelerates with a relatively larger deceleration magnitude. This enables us to evaluate how the platoon responds to such extreme scenarios. We present the platoon’s performance during this scenario in the Results section.

Vehicles publish their ROS messages at an average rate of ≈ 10 Hz-relatively slower than the suggested maximum frequency of 33 Hz, in European Telecommunications Standards Institute (2019), because we expect the signal transmission to the GPS receivers to be slower and, in some cases, unstable. We also configure the GPS with an update rate of 0.1 s. We only update feedback to the PID controllers when the corresponding GPS sensors publish new measurement data. We tune the GPS with the following settings: a standard deviation of the additive Gaussian noise to the position of 0.01 for the latitude, longitude, and altitude, the standard deviation of the relative velocity error in GPS readings is 0.1 m/s.

### Platoon model, longitudinal inter-vehicle distance control and problem statement

The controlled platoon comprises four vehicles in total. The Leader Vehicle (*LV*) and three Follower *F* vehicles. The platoon model considered in this study is based on the predecessor-leader following (PLF) communication model described by Swaroop & Hedrick (1999), Zheng et al. (2014) and Wang, Wu & Barth (2018). We design a PID controller to aid the control of the distance between vehicles. It takes as input the current inter-vehicle distance between vehicles (*d*_*i*_ to the preceding vehicle and *d*_*il*_ to the *LV*) and returns as output a velocity reference with which the corresponding *F* Vehicle needs to travel to achieve the desired distance (*D*) to the preceding vehicle and *D*_*il*_ to the *LV*.

**Figure 3:**
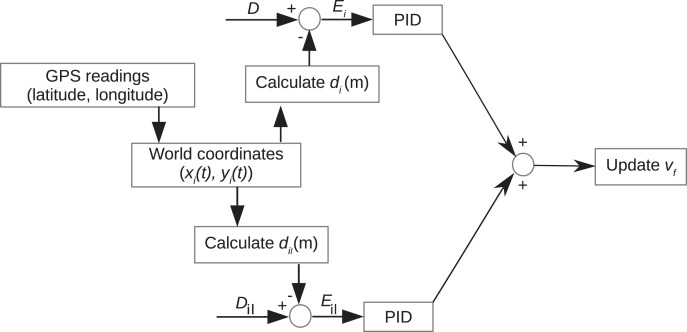
Platoon controller model.

Figure 3 depicts data acquisition, the specific parameters used by the PID algorithm, and how, ultimately, the reference velocity of the corresponding *F*_*i*_ vehicle is calculated from the joint outputs of the PID controllers where *x*_*i*_(*t*), *y*_*i*_(*t*) are the corresponding vehicle’s world coordinates.

We thus state the problem as:

(6) $$\matrix{ {Set({d_i}) = D \pm {E_i}} \hfill \cr {Set({d_{il}}) = {D_{il}} \pm {E_{il}}} \hfill \cr }$$

where,

(7) $$\matrix{ {{E_i} = D - {d_i}} \cr {{E_{il}} = {D_{il}} - {d_{il}}} }$$

${\rm and}\; {E_i} = {E_{il}} \leq {E_{thresh}}.$

Ultimately, the primary purpose of our PID controllers is to reduce the errors, *E*_*i*_ and *E*_*il*_ and drive them as close to 0 m as possible.

So the best-case scenario at any point in the simulation is to have *E*_*i*_ = *E*_*il*_ = 0 m, especially during the steady-state. For evaluation purposes, analyzing the results of *E*_*i*_ is sufficient since *E*_*i*_ is directly proportional to *E*_*il*_, that is, *E*_*i*_ = *k*· *E*_*il*_. Where *k* is the constant of proportionality. *k* = 1 in this case. Thus we omit values of *E*_*il*_ and, incidentally, *d*_*il*_ from the graphs and tables for brevity.

*∀ F*_*i*_, *i* ∈ {1, 2, 3} where *d*_*i*_ is the *i*^*th*^ inter-vehicle distance between the *i*^*th*^ *F* vehicle and the preceding vehicle, *d*_*il*_ is the inter-vehicle distance between the *i*^*th*^ *F* vehicle and the *LV*, *D* is the desired inter-vehicle distance between the *i*^*th*^ *F* vehicle and its corresponding predecessor, *D*_*il*_ is the desired inter-vehicle distance between the *i*^*th*^ *F* vehicle and the *LV*, *E*_*i*_ is the error between the *i*^*th*^ inter-vehicle distance and the desired distance, *D* and *E*_*il*_ is the error between *d*_*il*_ and *D*_*il*_. *E*_*thresh*_ is the maximum and minimum threshold value beyond which any of the errors should not exceed. This constraint ensures that *F* vehicles should not fall more than *E*_*thresh*_ behind the preceding vehicle, *i.e*., *E*_*i*_ ≤ *E*_*thresh*_ and *E*_*il*_ ≤ *E*_*thresh*_. It also ensures that *F* vehicles do not get more than *E*_*thresh*_ closer to the preceding vehicle and, incidentally, the *LV*, *i.e*., *E*_*i*_ ≥ − *E*_*thresh*_ and *E*_*il*_ ≥ − *E*_*thresh*_. Every *F* vehicle runs the PID control algorithm.

Applying the platoon stability definition provided by Seiler, Pant & Hedrick (2004), the steady-state error transfer function can be written as

(8) $$H(s) = \displaystyle{{{E_i}} \over {{E_{i - 1}}}}$$

This implies that platoon stability is locally guaranteed under the condition that ||*H*(*s*)||_*∞*_ ≤ 1, and *h*(*t*) > 0 where *h*(*t*) yields the impulse response corresponding to *H*(*s*) as per the *ζ*_2_ norm as defined by Öncü et al. (2012). *ζ*_*∞*_ extends this notion throughout the whole platoon to ensure that overshoots do not occur as the signals propagate up the string, hence global stability.

**Figure 4:**
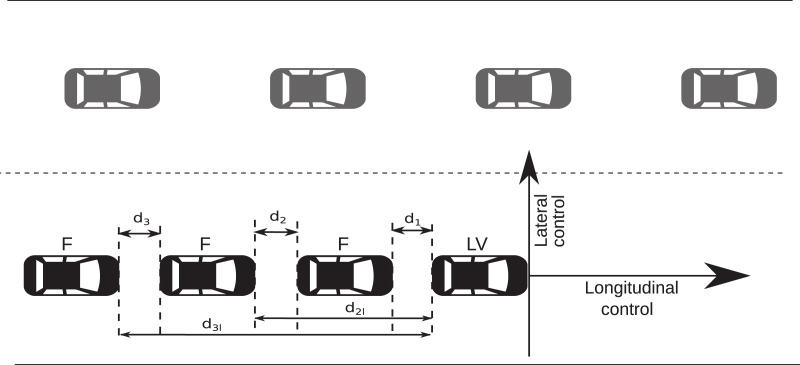
Illustration of the inter-vehicle distance, *d_i_*, *d_il_*, *LV*, and *F*.

Figure 4 demonstrates the platoon setup. *LV*, is the Leading vehicle, also referred to as the root node of the platoon, and is generally indexed as the first member of the platoon. The *F* labels depict the follower vehicles. The distance from one vehicle’s head to the preceding or LV vehicle’s rear is referred to as the inter-vehicle gap/distance in this study.

The control algorithm aims at ascertaining a constant inter-vehicle gap with all vehicles’ velocity sufficiently approximately equal to that of the *LV* in the platoon using only the distance calculated from the data provided by the onboard GPS sensors of the vehicles. Errors in the inter-vehicle gap should be bounded, and there should be no collisions among platoon members in the worst-case scenario at all times during the simulation. In the simulation, constraint parameters were set as; *E*_*thresh*_ = 5 m, *D* = 7 m.

At the start of the simulation, GPS data measurements are retrieved asynchronously if available from every vehicle’s onboard GPS sensors. From this data, the relative inter-vehicle gap is calculated and forwarded to the PID algorithm, which in turn returns the reference velocity with which the corresponding *F* vehicle’s velocity is updated with the aim to achieve the desired inter-vehicle gap between *F* and the preceding vehicle. The desired inter-vehicle distance, *D*, is set as the PID algorithm’s *setpoint*, whereas the inter-vehicle gap estimate, calculated from the data measurements provided by the GPS sensors, is provided as the *feedback* for the PID control algorithm. Similarly, the inter-vehicle gap between a given *F* vehicle and *LV*, with the exception of the second *F* vehicle, is controlled using *d*_*il*_ as the feedback and *D*_*l*_ as the setpoint of the PID. We repeat these steps throughout the simulation lifetime.

## Results

We performed simulations by periodically varying the *LV’s* reference velocity seamlessly to *V*_1_ = 22 m/s, *V*_2_ = 10 m/s and *V*_3_ = 5 m/s. We do this by publishing velocity commands to the *LV*’*s* velocity topics *via* ROS. Reference velocities of the *F* vehicles were determined by the PID controllers. The desired inter-vehicle distance, *D*, in the simulations was set to 7 m. *V*_1_, *V*_2_, *V*_3_, and *D* were arbitrarily chosen. Table 2 shows the PID gains used by the PID controllers.

**Table 2:** PID controller parameters.

Parameter	Value
*P*	0.07
*I*	0.00005
*D*	0.08

**Figure 5:**
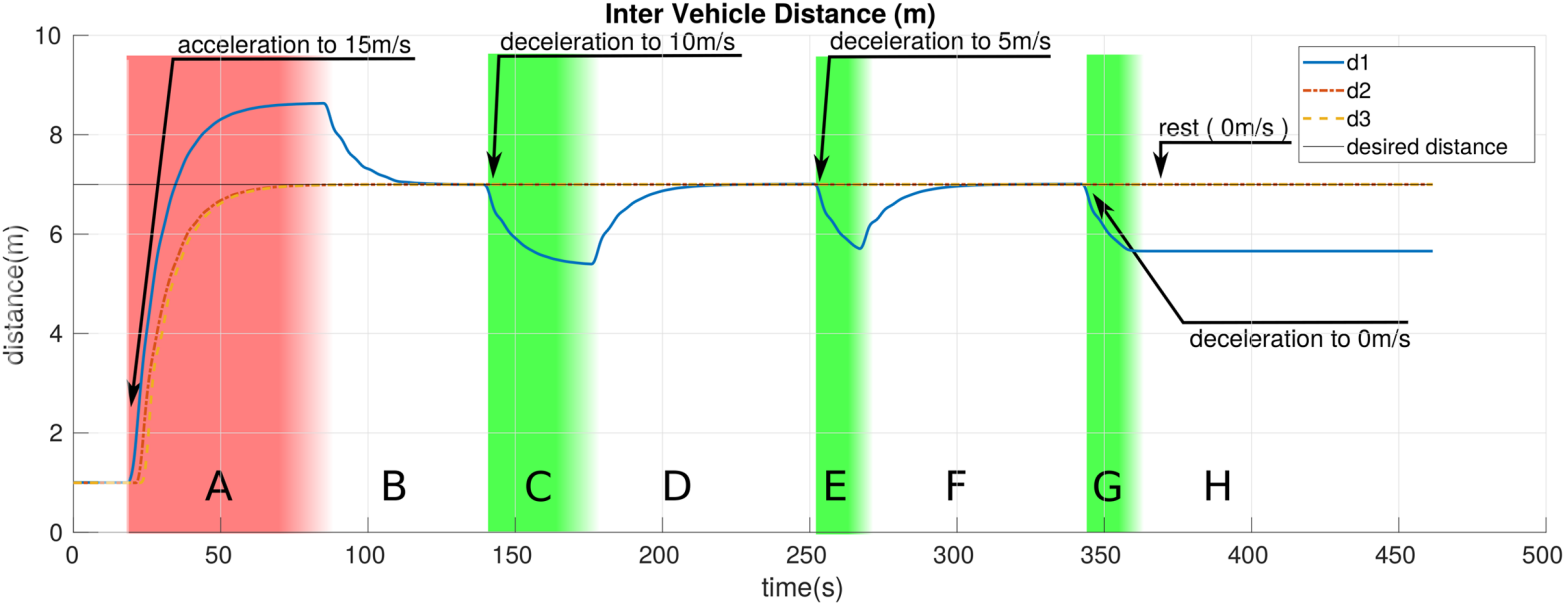
The inter-vehicle distance of platoon members over time.

**Figure 6:**
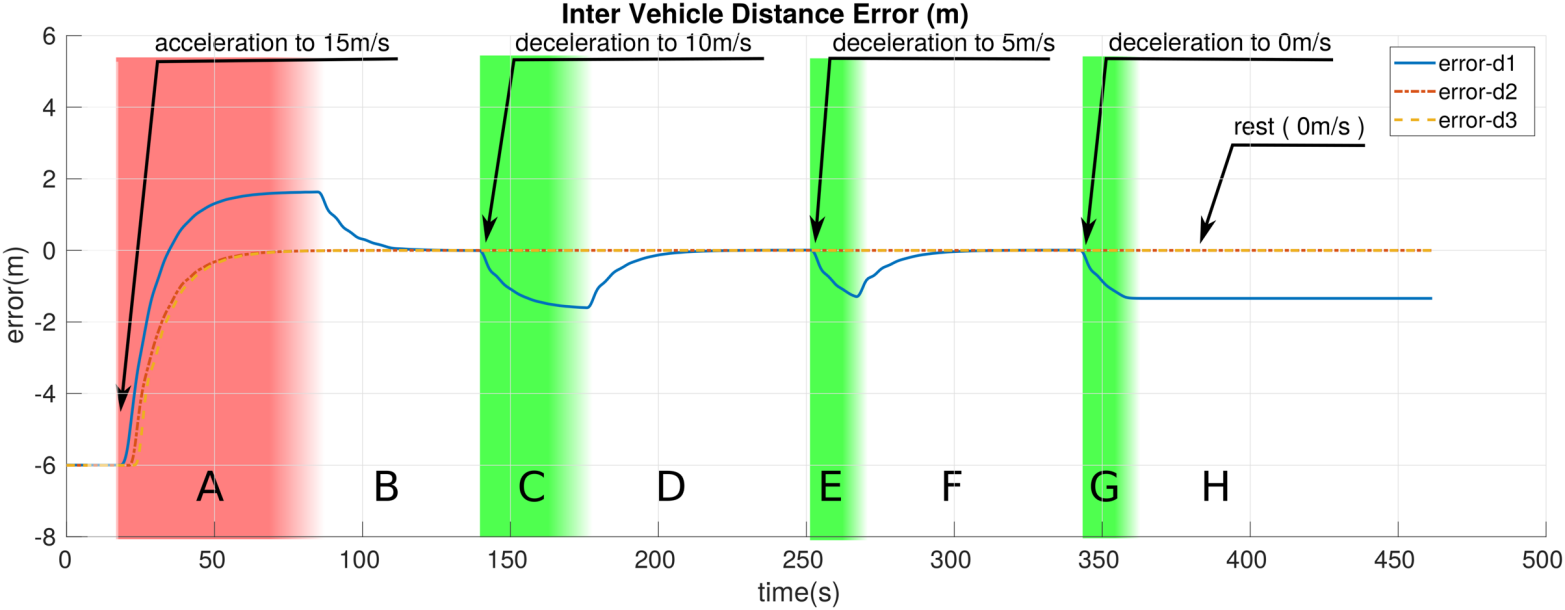
The error in the inter-vehicle distance of the platoon members.

**Figure 7:**
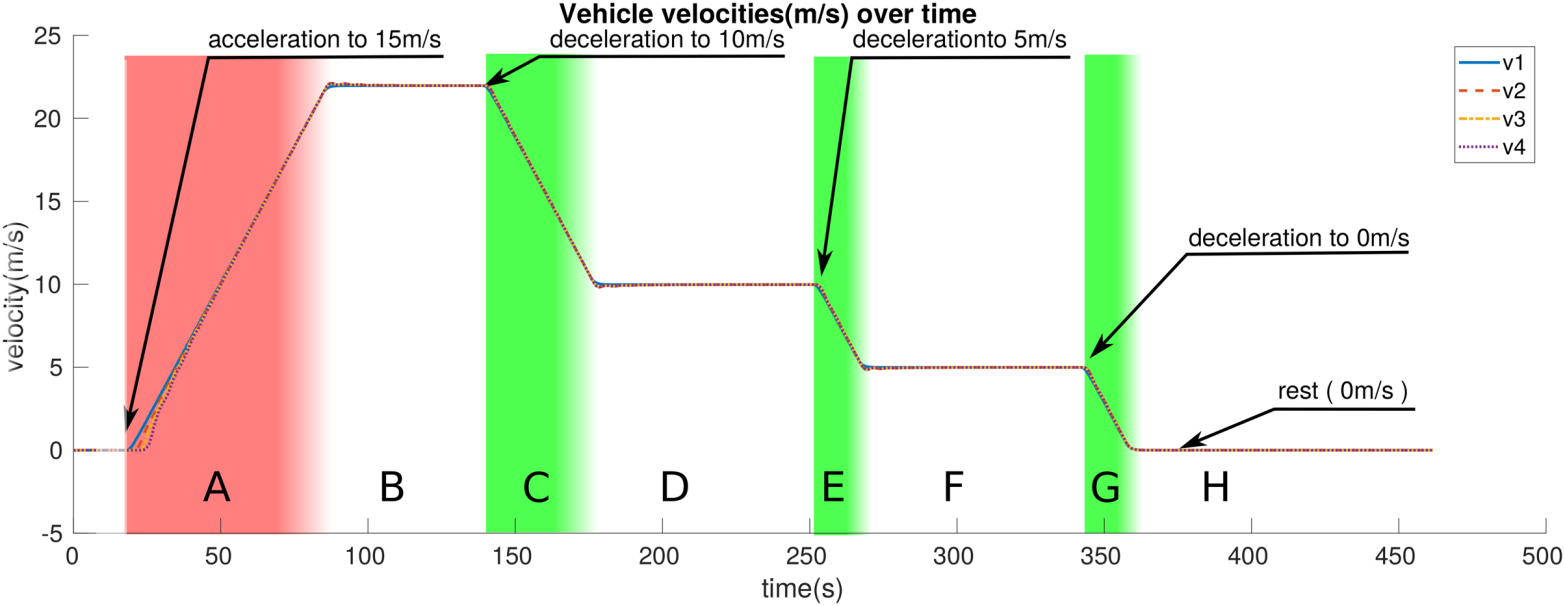
The velocity of platoon members over time.

We break down the simulation and entire motion of the platoon into eight distinct zones represented by letters *A* through *H* in Figs. 5–7. These zones are different color-coded for a more straightforward distinction. These colors, in brief, represent: red: the zone where *LV* is accelerating; white: zone where the *LV* is moving with constant velocity or at rest; green: zone in which the *LV* is decelerating. Table 3 provides a more detailed description of the zones. This section presents the results obtained from the simulations.

**Table 3:** Description of the distinct zones throughout the simulation.

Zone	Color	Explanation
*A*	Red	*LV* Accelerating from 0 to 22 m/s
*B*	White	*LV* moving with constant velocity, 22 m/s
*C*	Green	*LV* decelerating from 15 to 10 m/s
*D*	White	*LV* moving with constant velocity, 10 m/s
*E*	Green	*LV* decelerating from 10 to 5 m/s
*F*	White	*LV* moving with constant velocity, 5 m/s
*G*	Green	*LV* decelerating from 5 m/s to rest
*H*	White	All vehicles at rest with 0 m/s.

Figure 5 shows the change in the inter-vehicle distance of platoon members over simulation time.

Figure 6 shows how the corresponding error in the inter-vehicle distance between the platoon members changes over time during the simulation in reference to the desired inter-vehicle distance. It can be noted that *F*_1_ generally experiences much greater errors in comparison to *F*_2_ and *F*_3_ as it only gets information from one vehicle (*LV*) whereas *F*_2_ and *F*_3_ receive information from two different sources (preceding vehicle and *LV*).

Figure 7 presents the velocity of the platoon members throughout the simulation.

We manually tuned the PID controller. The parameter gains of the controller that yielded the presented results are listed in Table 2. These parameters were selected by trial and error.

**Figure 8:**
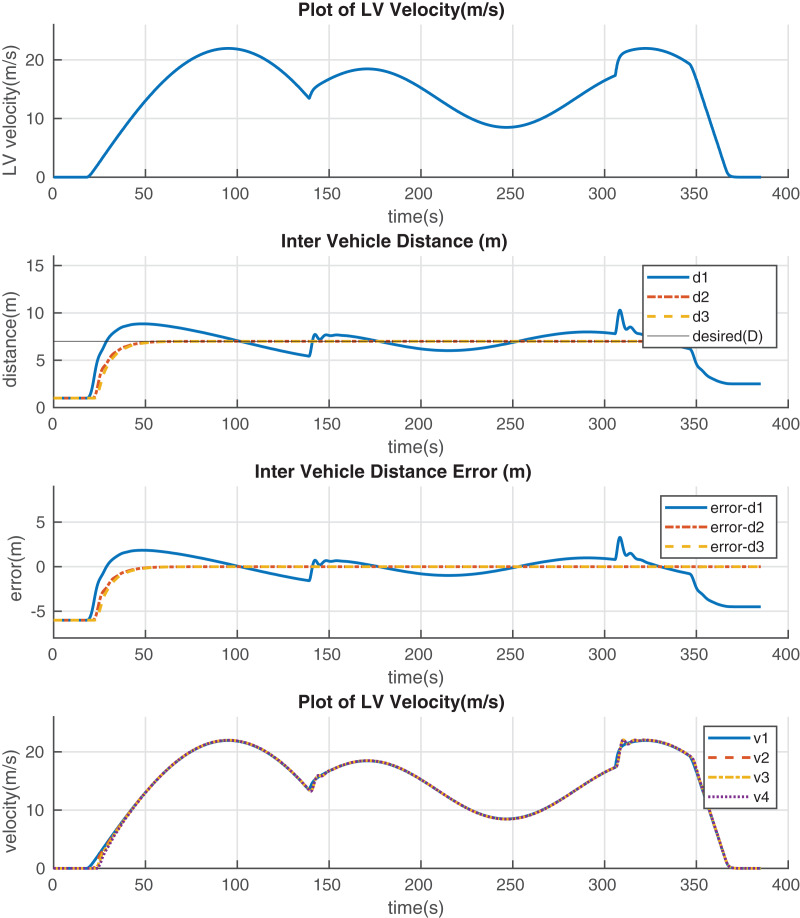
Platoon performance when LV velocity is continuously varied-uncertain scenario.

Table 4 provides statistics of the error in the inter-vehicle distances at different zones throughout the simulation. The statistics presented are the standard deviation (std) and the variance (var) of the inter-vehicle distances within the specified regions. Figure 8 shows the results of the proposed controller’s performance when the *LV*’*s* velocity is continuously changing. From top to bottom, in Fig. 8, is the *LV’s* velocity plot throughout the scenario, followed by the platoon’s inter-vehicle distance, error, and velocity. The maximum and minimum observed errors during this scenario are 3.30 m and −4.50 m, respectively. The maximum acceleration magnitude observed in this scenario was 2 m/s^2^ observed when the elapsed time was 306 s. The LV accelerated from 17 m/s to 21 m/s in 2 s. It can also be observed that the LV decelerates relatively fastly towards the end of the scenario till it comes to rest. That is, from the period when the elapsed time is 348 s till the LV comes to rest in Fig. 8. The *LV* decelerates from 19.5 m/s to 0 m/s in 17 s. It should be noted that the error measurements used in the calculation of these statistics are those values logged after the first *F* vehicle observes an inter-vehicle gap of 7 m to the *LV* for the first time. That is, *d*_*i*_ = 7 m for the first time. Error values prior to this time are not taken into account during the calculation of statistics in order to mitigate the influence of the initial conditions to the performance evaulation of the proposed controller. The initial conditions, that is, all vehicles being 1 m apart were set arbitrarily. This serves two purposes, *i.e*., ensures that there is no reverse motion in the platoon and also enables us to monitor how well and how fast the system recovers from an inital error situation. The error plots, therefore, register starting error values of −6 m in all the inter vehicle distances, *d*_*i*_, between vehicles. The negative sign means that the vehicles are 6 m closer to each other than desired inter-vehicle distance. A positive sign in the error thus means that the vehicles are further from each other than desired.

**Table 4:** Error statistics of the three inter-vehicle distances.

*d*_*i*	*d*_1		*d*_2		*d*_3	
Zone	std (m)	var (m^2^)	std (m)	var (m^2^)	std (m)	var (m^2^)
*A*	0.3876	0.1502	0.3701	0.1370	0.4344	0.1887
*B*	0.2410	0.0581	0.0014	0.0000	0.0024	0.0000
*C*	0.3890	0.1513	0.0006	0.0000	0.0002	0.0000
*D*	0.2359	0.0557	0.0000	0.0000	0.0001	0.0000
*E*	0.4284	0.1835	0.0013	0.0000	0.0001	0.0000
*F*	0.2028	0.0411	0.0002	0.0000	0.0001	0.0000
*G*	0.4737	0.2244	0.0001	0.0000	0.0001	0.0000

Table 5 presents the comparison of our controller’s performance with that of a different approach proposed by Ali, Garcia & Martinet (2013). In Ali, Garcia & Martinet (2013), suggest a variable inter-vehicle distance control approach where they define the time headway term proportional to the difference between the velocity of the vehicle, and an additional term, hereafter, referred to as *ρ*, which is shared by all platoon members. Thus, the spacing error is evaluated. The additional term, *ρ*, is also used as a control parameter for their modified constant time headway (CTH) algorithm. Ali et al.’s control approach uses both *local and global control*. In Table 5, local control is represented by *l*, whereas global control is represented by *g*. In local control, data from neighboring platoon members are utilized, whereas in global control, data from, at the very least, the *LV* is necessary. We use global and distributed control in our approach. To evaluate the performance of their approach, they vary the velocity of the *LV* three times to *V*_1_′ ≈ 14 m/s, *V*_2_′ ≈ 19 m/s, and *V*_3_′ ≈ 16 m/s. They perform simulations with both MATLAB and TORCS. MATLAB is used to represent a perfect world, and TORCS, a 3D, simulation environment, is used to represent a rather more realistic environment since it provides more features. We utilize the results obtained using the rather more realistic TORCS environment in the comparison provided in Table 5. Requiring extra information and parameters from other platoon members increases the computation overhead of the algorithm. In Table 5, we present the comparison of the maximum transient error, the steady-state error, and the communication criteria (*Com*.) used by the two different approaches. Long, Tian & Cheng (2020) proposed a distributed model predictive model for the longitudinal control of truck platoons. Their model considers the state of the *LV*. In their study, platoon members transition from cruise control (CC), adaptive cruise control (ACC), and cooperative cruise control (CACC). ACC and CACC constitute the most advanced platoon controllers that exist today. They use MATLAB to evaluate the performance of their model. We present a summarized comparison of the performance of our approach and the ACC phase of their study. Throughout the simulations, Long, Tian & Cheng (2020) had the *LV* accelerate to ≈ 16 m/s, then move with constant velocity after that. They experienced a maximum transient response error of ≈ 37 m before the system finally settled and had the range error converge to 0 m. Our proposed controller had a maximum transient error of 1.6322 m, after which it converged to 0 m at steady-state.

**Table 5:** Comparison of results with another proposed approach.

Method	Max transient	Steady-state error (m)			Com.
	error (m)	*V*_1_	*V*_2_	*V*_3_	
Our method	1.6322	0	0	0	*l* & *g*
Ali, Garcia & Martinet (2013)	≈ 3.2	≈ 1.8	≈ 2.5	≈ 2.2	*l* & *g*

## Discussion

In this study, we provide a computationally less demanding longitudinal inter-vehicle distance control algorithm for a platoon of autonomous vehicles that only requires that vehicles be equipped with GPS sensors and have a connection to Wi-Fi. There are two major categories of platoon control: centralized (global) and decentralized (local). In the centralized control, a single unit is responsible for the main data processing, control calculations and the transmission of control commands to the platoon members. In the decentralized control, every platoon member handles its own data processing and runs its control algorithm. The proposed approach is relatively more computationally cost-efficient because the controller presented in our approach is decentralized. Each vehicle handles its own processing of the data retrieved from the GPS sensor unlike in approaches based on centralized control (Milutinovic & Lima, 2006). In our proposed approach, no matter how large (in terms of number of platoon members) the platoon becomes, data processing and running the control algorithm costs same unlike centralized approaches. This approach thus scales with platoon size. Data processing requirements do not change as the number of vehicles in the platoon grows unlike in centralized setups for instance in Milutinovic & Lima (2006), Sacone et al. (2021) where relatively more powerful calculation resources are required at the main processing unit.

Additionally, the proposed approach is financially cost-efficient and relatively simpler to implement yet providing considerably good real-time performance. All that is required for this algorithm is a single GPS sensor per platoon member. This is relatively much simpler to setup and configure in comparison to sensor fusion approaches that require a minimum of two sensors (*e.g.*, GPS and IMU) such as the one presented by Sukkarieh, Nebot & Durrant-Whyte (1999). Setting up, calibrating and configuring sensors in a sensor fusion system is relatively more complex, financially and computationally more resource demanding as well. It can be observed from the results provided that the proposed algorithm guarantees the following effect in the platoon while maintaining the desired inter-vehicle distance. The algorithm also ensures platoon formation preservation and no collisions amongst platoon members since the error, *E*_*i*_ ≥ − *E*_*thresh*_ throughout the simulation. The algorithm computation cost is less than most control algorithms described in the literature. For example, physics-inspired control algorithms such as Yi & Chong (2005), most of the other PID-based algorithms with different approaches like taking as input to the PID the preceding vehicle’s velocity and acceleration. For instance, strategies described by Ioannou & Xu (1994), Sivaji & Sailaja (2013), and Ali Memon, Jumani & Larik (2012). The PID-based technique suggested by Villagra et al. (2010) generates inter-vehicle distances between vehicles using a dynamic model, whereas our approach does not need a model to generate the inter-vehicle distances. All inter-vehicle distances are obtained from measurements taken by the vehicle onboard GPS sensors, thereby using less computation resources. Thus, extra computing resources reserved for longitudinal inter-vehicle gap control can be allocated to more demanding platoon applications. Furthermore, this study differs from those that only perform numeric simulations such as Sivaji & Sailaja (2013), Zheng et al. (2014) and Ali Memon, Jumani & Larik (2012) by not only using generated GPS data but also by applying the algorithm to the 3D models of the platoon to mimic the real world as much as possible.

Furthermore, following from the definition of string stability provided by Öncü et al. (2012), string stability is guaranteed in this study since overshoots do not occur as signals propagate up the string. Ignoring the initial errors culminating from initial conditions, string stability can be proven using eight almost everywhere.

However, our approach does have limitations. The first limitation of this algorithm stems from the fact that it is mainly based on GPS sensors. GPS, in reality, is affected by high-frequency faults culminating from multipath errors that occur when signals bounce off surfaces before they can reach the sensor receivers. The position fix, therefore, gets affected as the signals are delayed. Another rarer cause of GPS faults happens when one of the satellites used by the sensor receiver gets blocked and, as a result, has to be compensated by signals received from a different satellite. The position fix received by the GPS sensor is affected by the geometry of the satellites from which the sensor gets signals. So, such changes in configurations of the satellite observed by the sensor receiver affect the position fix finally reported by the GPS receiver.

High-frequency faults and multipath make the accuracy of GPS sensor, and ultimately, the efficiency of our algorithm heavily environment-dependent, making it more accurate and preferable in open space areas than in underground passages, enclosed environments, or places with tall buildings such as skyscrapers. The algorithm can be incorporated into indoor environments or closed environments by replacing the GPS technology with higher precision localization tools and, or sensors such as beacon technology illustrated by Surian et al. (2019) and Siegwart & Nourbakhsh (2004). We meticulously analyzed the impact of factors such as delays in V2V communication, delays in signal propagation from the PID controllers to the vehicle acuators on the performance of the proposed approach. We also analyzed the impact of GPS sensor lags and the random abrupt acceleration or deceleration of the LV due to miscellaneous factors such as having to stop at the traffic lights, inaccuracies resulting from following a human controlled vehicle and the like in Gunagwera & Zengin (2022). Gunagwera & Zengin (2022) provided graphical results along with quantitative analysis of their study.

Autonomous vehicle platooning applications in urban areas involve high precision dependent maneuvers that require about 0.02 m accuracy to guarantee safety, among other requirements - such as lane-keeping/changing on busy streets, overtaking operations, to mention but a few. In such applications, a 0.5 m error is pretty significant. Our results show that the transient errors increase with increasing velocity, thereby decreasing the overall accuracy of the proposed approach. This limitation will be rectified in our future works, as well.

Thus, expanding the applicability of the proposed algorithm to all types of roads and environments requires fusing data from other sensors such as LIDAR, the Inertial Measurement Unit (IMU), and camera. Using sensor fusion to enhance the applicability of the proposed algorithm and make it more ideal for even more complex environments is one of our future studies.

## Conclusion

In this study, we present a decentralized algorithm to control the longitudinal inter-vehicle distance of a platoon. In the presented approach, each vehicle handles its own data processing and the running of the control algorithm. Data required by the control algorithm is provided by a single GPS sensor, thus, making the proposed algorithm cost-efficient both financially and computationally. The proposed approach takes as input to the PID controller, the updated inter-vehicle distance between a follower vehicle and the preceding and/or leader vehicle. This distance is calculated from the data measured and provided by the vehicles’ onboard GPS sensors. The controller returns the reference velocity with which the follower vehicle should move to achieve the desired inter-vehicle distance. 3D simulations using Gazebo and ROS are additionally used to verify and monitor the performance of the system. The proposed approach guarantees the following effect of the platoon ensuring maintenance of platoon formation and no collisions among platoon members. Furthermore, after the transient response to the leader vehicle’s acceleration, the standard deviation of the inter-vehicle distance error was kept under 7% of the desired inter-vehicle distance throughout the entire simulation period. The system achieved a 0 m error at the steady-state when the LV moves with constant velocity. However, the proposed method is mainly suitable for open environments since GPS accuracy is susceptible to high-frequency errors resulting from multipath and collision of GPS signals with surfaces before they reach the receiver. Applicability of the approach can be extended to closed and underground environments if GPS is replaced with high precision localization equipment such as position beacons installed in the target environments. We are currently working on expanding the operability and applicability of the proposed approach on more road types, environments (urban, rural, to mention but a few). Incorporating more sensors and sensor fusion techniques to improve the accuracy of not only the inter-vehicle distance but also the velocity of the platoon members is another direction for our future work.

## Supplemental Information

10.7717/peerj-cs.990/supp-1 Supplemental Information 1 Core code for the performed simulations.

## Additional Information and Declarations

### Data Availability

The following information was supplied regarding data availability: The core code used to perform the simulations is available in the Supplemental File.
